# Eye structure, activity rhythms, and visually-driven behavior are tuned to visual niche in ants

**DOI:** 10.3389/fnbeh.2014.00205

**Published:** 2014-06-13

**Authors:** Ayse Yilmaz, Volkan Aksoy, Yilmaz Camlitepe, Martin Giurfa

**Affiliations:** ^1^Department of Biology, Faculty of Sciences, Trakya UniversityEdirne, Turkey; ^2^Department of Behavioral Physiology and Sociobiology, University of WürzburgWürzburg, Germany; ^3^Research Centre for Animal Cognition, Université de ToulouseToulouse, France; ^4^CNRS, Research Centre for Animal CognitionToulouse, France

**Keywords:** compound eye, activity rhythm, visual learning, ant, *Camponotus aethiops*, *Formica cunicularia*

## Abstract

Insects have evolved physiological adaptations and behavioral strategies that allow them to cope with a broad spectrum of environmental challenges and contribute to their evolutionary success. Visual performance plays a key role in this success. Correlates between life style and eye organization have been reported in various insect species. Yet, if and how visual ecology translates effectively into different visual discrimination and learning capabilities has been less explored. Here we report results from optical and behavioral analyses performed in two sympatric ant species, *Formica cunicularia* and *Camponotus aethiops.* We show that the former are diurnal while the latter are cathemeral. Accordingly, *F. cunicularia* workers present compound eyes with higher resolution, while *C. aethiops* workers exhibit eyes with lower resolution but higher sensitivity. The discrimination and learning of visual stimuli differs significantly between these species in controlled dual-choice experiments: discrimination learning of small-field visual stimuli is achieved by *F. cunicularia* but not by *C. aethiops*, while both species master the discrimination of large-field visual stimuli. Our work thus provides a paradigmatic example about how timing of foraging activities and visual environment match the organization of compound eyes and visually-driven behavior. This correspondence underlines the relevance of an ecological/evolutionary framework for analyses in behavioral neuroscience.

## Introduction

Social insects constitute an established model for the study of visual perception and learning and have contributed important insights into the principles of vision and visual cognition (Menzel and Backhaus, 1991; Dafni et al., 1997; Giurfa and Menzel, 1997; Briscoe and Chittka, 2001; Srinivasan, 2010; Avarguès-Weber et al., 2011; Dyer, 2012; Zhang et al., 2012). Among social insects, ants offer excellent opportunities for cross-species analyses of visual performance and architectures (Menzi, 1987; Greiner et al., 2007; Narendra et al., 2011), due to their high ecological diversity and species-richness (Hölldobler and Wilson, 1990). Ant species may differ in their visual niches and light conditions and thus experience different selective pressures on their visual systems. As a consequence, their compound eyes may exhibit adaptations to specific life styles (Greiner et al., 2007; Narendra et al., 2011) where trade-offs between spatial resolution and sensitivity are expected (Kirschfeld, 1976; Land, 1997). These trade-offs may, in turn, affect visually-driven behaviors.

**Figure 1:**
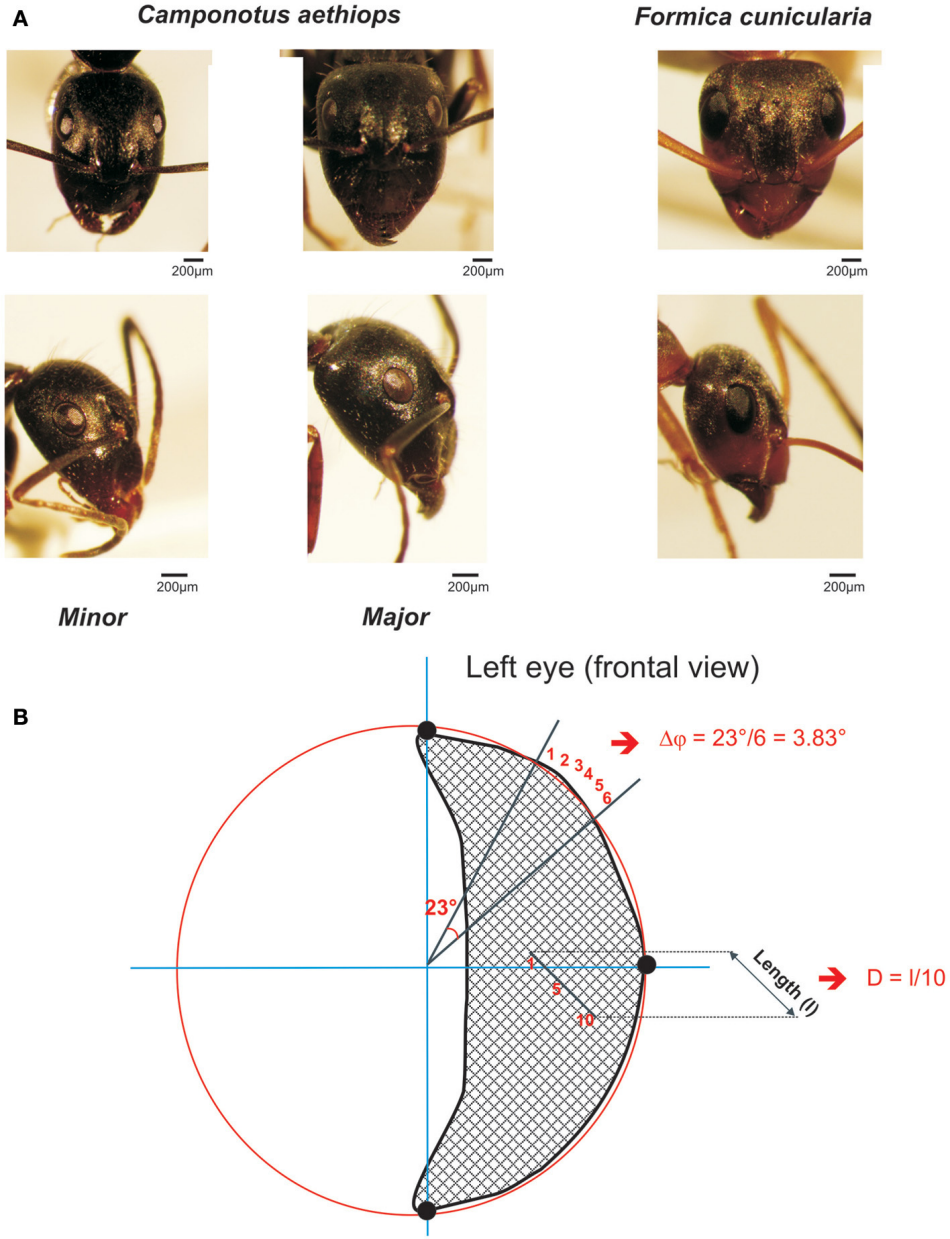
**The two sympatric ant species used in this study. (A)** Frontal (upper row) and lateral views (lower row) of the heads and compound eye of *Camponotus aethiops* majors and minors and *Formica cunicularia*. The scale is indicated in each case. **(B)** Drawing of a frontal view of a left compound eye to illustrate the calculation procedure for the *interommatidial angle* (Δφ) and the *ommatidial diameter*. Using the Image J software, three points (black dots) were defined on the outer visible perimeter of the eye and a circle (in red) was drawn to connect them. Two lines, one horizontal and the other vertical (in blue), that ran through the center of the circle were also drawn. In this figure the *interommatidial angle* (Δφ) was calculated by dividing the angle of 23° defined by two lines (black) going from the perimeter to the center of the eye by the number of ommatidia (6) on the perimeter section (i.e., Δφ = 3.83 ° in this example). The *ommatidial diameter* was measured by drawing and measuring the length (l) of a line going through a row of 10 ommatidia and dividing that length by 10, the number of ommatidia crossed.

We studied two sympatric ant species in the northwest of Turkey, which possess apposition eyes but differ in the characteristics of their visual environments: *Formica cunicularia* ants are considered to be diurnal, while *Camponotus aethiops* ants are reported to be rather crepuscular and nocturnal (Figure 1A). Diurnal ants foraging for food during daylight hours are not confronted with limited light. Crepuscular and nocturnal ants, on the contrary, are subjected to the problem of seeing at low levels of illumination and may sacrifice visual accuracy in order to capture more light (Warrant and McIntyre, 1990; Land, 1997). To test these hypotheses using *F. cunicularia* and *C. aethiops* as models, we quantified their behavioral rhythms, performed an analysis of their eye parameters, and studied their visual pattern discrimination in controlled laboratory conditions. We provide in this way an integrative analysis of the interplay between visual niche, lifestyle (Land, 1997; Van Hateren, 1997), morphological variations in eye organization (Land and Fernald, 1992; Warrant and McIntyre, 1993), and visual discrimination capacities.

## Materials and methods

### Activity rhythms

We monitored visually the activity rhythms of two sympatric nests, one of *F. cunicularia* and the other of *C. aethiops*. Both were located in the Güllapoğlu Arboretum of Trakya University, Edirne, Turkey (latitude: 41° 40′ 0" N, longitude: 26° 34′ 0" E). After defining an imaginary circle of 25-cm diameter around each nest entrance, we counted inbound and outbound worker ants on a 20 h basis (from 5:00 AM to 01:00 AM of the next day) during 10 days (May 2011). Counting of ants was performed during intervals of 3 min for *F. cunicularia*, and of 5 min for *C. aethiops* (owing to their lower activity), with 2 h spacing between consecutive measurements (11 measurements per day in total). In the case of the latter species, which is polymorph, separated counting was performed for *minor*- and *major* workers. Due to the short counting period, our goal was to provide a snapshot of the ants' activity in terms of the number of individuals present in the counting area at a given time of the day.

Temperature and illumination were measured in parallel at the nest entrances using a digital thermometer (RMR202, Oregon Scientific, Neotech Teknolojik Ürünler Dağıtım A.Ş., Turkey) and a digital light-meter (CHY-332; Centenary Materials, Taiwan), respectively.

### Allometry and eye structure

We measured the length and width of the compound eyes (C_*l*_ and C_*w*_, respectively) and the length of the thorax (Th_*l*_) (both in mm) in *F. cunicularia* and in majors and minors of *C. aethiops*. Compound eye structure was characterized by means of corneal replicas obtained from a thin layer of nail polish applied to the eye (Narendra et al., 2011). After drying, the polish was removed and photographed under light microscope (Olympus BH-02) equipped with a digital camera (Progress C12 Laser Optic System). Images were then digitized in a computer for quantification of the *total number of ommatidia* (TO), which was obtained from a direct count on a lateral view of the eye (Narendra et al., 2013). The total surface of the compound eye (A, μm^2^) was calculated using the formula for the area A of an ellipse with length C_*l*_ and width C_*w*_ (Moser et al., 2004) as:

$$A\text{=}\;\pi \left[\frac{Cl}{2}\times \frac{Cw}{2}\right]$$

To this end, length C_*l*_ and width C_*w*_of the compound eye were measured using the Image J software (National Institute of Mental Health, Bethesda Maryland, USA).

To quantify the *mean interommatidial angle* (Δφ), which describes the cornea sampling density and the *mean ommatidial diameter* (D) (μm), which provides a measure of the sensitivity to light (Land, 1997), we used frontal photographs of compound eyes (examples frontal views are shown in Figure 1A, upper row). On these photographs, Image J software allowed us to define three points on the outer visible perimeter of each eye (left or right) and draw a circle that connected them (see example in Figure 1B: the three black dots are connected by the red circle line). The perimeter of this circle included the eye's outer perimeter (Figure 1B). Two lines, one horizontal, and the other vertical, which ran through the center of the circle were drawn (see blue axes in Figure 1B).

To calculate the *interommatidial angle* (Δφ), we measured the number of ommatidia on the perimeter section comprised between two lines going from the perimeter to the center of the eye; the angle between these two lines was established and the interommatidial angle was obtained by dividing the angle yielded by the software by the number of ommatidia on the perimeter section (Döring and Spaethe, 2009; see Figure 1B). The *mean interommatidial* angle was obtained for each ant after repeating three times this calculation in different areas of the compound eye that were chosen randomly.

The *ommatidial diameter* was measured by drawing and measuring a line going through a row of 5–10 ommatidia in the horizontal or the vertical plane, and dividing that length by the number of ommatidia crossed (see Figure 1B). The *mean ommatidial diameter* was obtained for each ant after repeating three times this calculation in different areas of the compound eye that were chosen randomly.

In addition, the eye parameter (P), which allows examining the trade-off between sensitivity and resolution of a compound eye (Snyder, 1979), was obtained by multiplying the mean interommatidial angle (Δφ) by the mean ommatidial diameter (D)(Snyder, 1979) as:

P = D. Δ φ

### Visual discrimination learning

**Figure 2:**
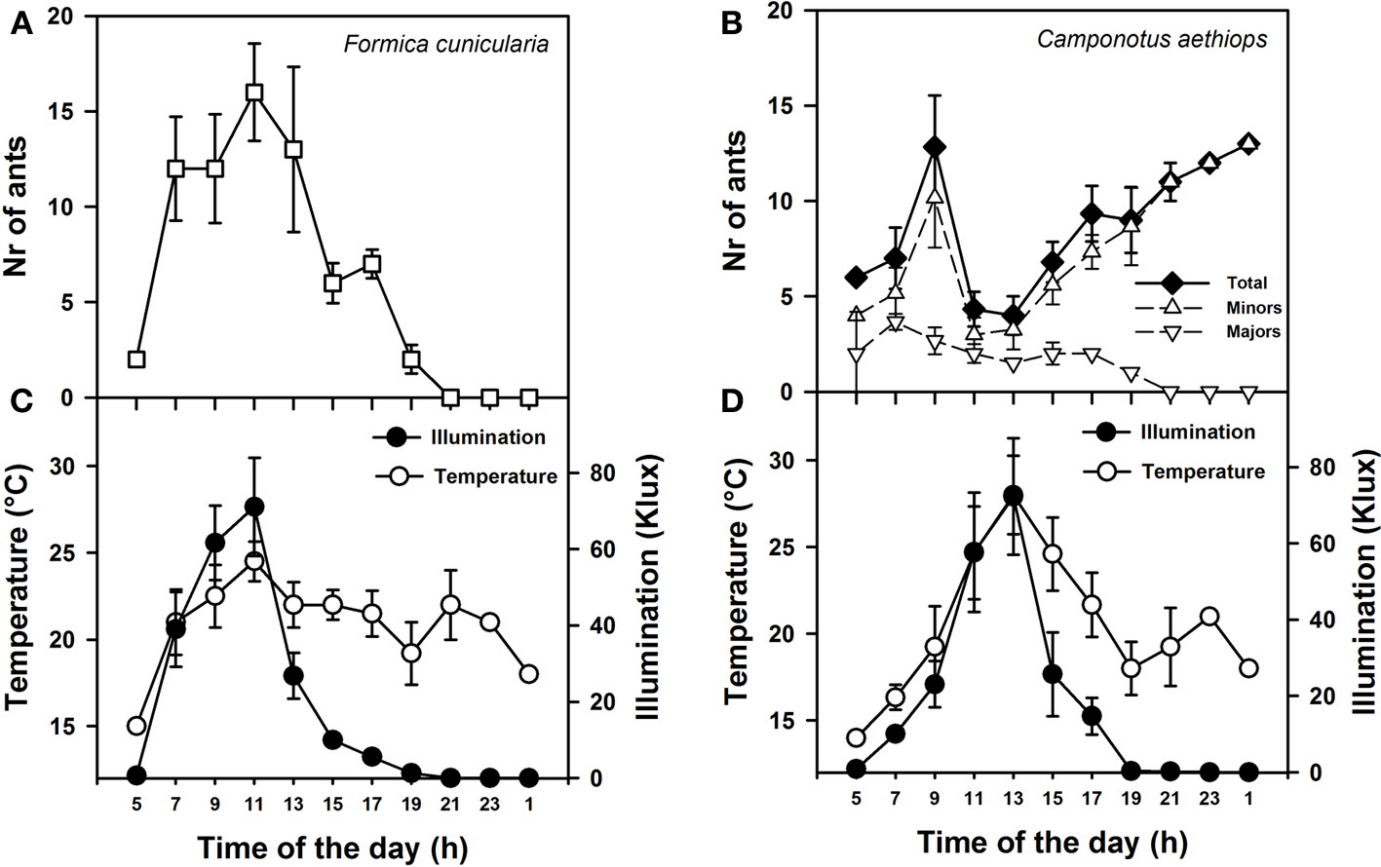
**Activity rhythms of *F. cunicularia* and *C. aethiops* as a function of environmental daylight and temperature. (A)** Activity rhythm of *F. Cunicularia*. **(B)** Activity rhythms of *C. aethiops* majors, minors and the sum of both (Total) are shown. **(C)** Temperature and daylight illumination measured in the immediate surroundings of *F. cunicularia* nest. **(D)** Temperature and daylight illumination measured in the immediate surroundings of *C. aethiops* nest.

We used an appetitive conditioning protocol to assess visual-stimulus discrimination in both ant species. In the case of *C. aethiops*, only minors were used in these experiments because they engage more in foraging activities than majors (Laffort et al., 1991; see Figure 2B).

Colonies were transferred to the laboratory with their original nest material and placed in plastic containers under constant laboratory conditions (50% relative humidity, 23–25°C, and 12 h/12 h light-dark regimen). Silicon pipes connected each nest to a feeding box, which the ants could freely visit. Diluted honey and dead insects were provided as carbohydrate and protein sources. Honey was removed from the box one month before the experiments to ensure high motivation for foraging.

#### Experimental setup

Marked ants were trained individually to enter a Plexiglas® Y-maze (Figure 5A) to collect 25% (weight/weight) sucrose solution (2 μl) on a rewarding visual stimulus that had to be discriminated from another visual stimulus punished with 5% quinine solution (2 μl). Both kinds of reinforcements have been shown to induce successful differential conditioning in ants learning olfactory discriminations in a similar maze (Dupuy et al., 2006; Josens et al., 2009). During training, the rewarded and punished stimuli were swapped pseudo-randomly between the arms of the maze to prevent ants from forming a side preference, using odor cues or to prevent room cues from playing any role in the ants' choice (Harris et al., 2005; Dupuy et al., 2006; Josens et al., 2009). Only one ant was present in the maze at a time. Solutions were provided on transparent plastic pieces (1 × 2 cm) that were adjacent to visual stimuli. All three arms of the maze were 20 cm long, with a 4 × 4 cm cross section. Once the experimental ant entered in the maze, it had to choose between two arms that formed an angle of 120° at the decision point. The maze walls were made from black opaque acrylic mounted on a white Plexiglas® plate. The maze had no ceiling. Illumination was provided by a D65 lamp (6500 K) similar in spectrum to sunlight and yielding an overall intensity of 1600 lux at the maze level, which corresponds to a partially overcast day (1000–2000 lux). Experiments were performed at a time of the day when appetitive behavior of both species was comparable.

#### Visual stimuli

Ants were trained to discriminate two small-field and two large-field visual stimuli. The former consisted of small black triangles (4 cm height and 4 cm base), one pointing upward and the other pointing downward, presented on a white background. Triangles were placed at a distance of 4.5 cm from the decision point of the maze and subtended a visual angle (vertical extent) of 47.9° at the decision point. The large-field stimuli were two large black-and-white gratings (35 × 40 cm), one horizontal, and the other vertical, which exceeded the extent of the maze back walls and were placed 20 cm from the decision point of the maze. Gratings had stripes 8 cm in width, each subtending a visual angle of 20.61°; an entire pattern subtended 82 × 90°. Although the ants did probably not perceive the whole pattern extent due to the presence of the lateral maze walls, they could definitely extend their visual sampling beyond the cross section of an arm's maze due to the absence of ceiling. As a consequence, even with limitations, the gratings offered a much larger area than the small triangles. Prior experiments with ants making visual discriminations in a maze have also used large-field patterns, which can be well-discriminated (Harris et al., 2005; Riabinina et al., 2011). Gratings were randomly shifted from visit to visit to prevent the exclusive learning of local cues. Horizontal gratings were shifted randomly upward and downward while vertical gratings were shifted rightward and leftward; in this way ants had to discriminate between either two white or two black areas on the back walls, thus forcing them to extend their visual sampling beyond the maze. Gratings also could be positioned with a half period centered on either back wall, thus displaying a vertical edge in one arm and a horizontal edge in the other arm.

#### Pre-training and training procedures

During a pre-training phase, ants were individually familiarized with the setup during four visits in which they learned to collect a drop of sucrose solution placed at the entrance arm, at the decision point (intersection of both lateral arms), and at each of the lateral arms of the maze in the absence of visual stimuli (Dupuy et al., 2006; Josens et al., 2009). Motivated ants that returned to the setup in less than 3–4 min were conserved for the training phase, which consisted of 18 consecutive visits to the maze (trials). Thus, the interval between consecutive training trials varied between 2 and 4 min. The 18 trials were grouped into three blocks of six trials each, in order to analyze the learning performance during the training. Based on the first choice performed within each training trial, an acquisition curve was established, which shows the proportion of correct choices along three consecutive training blocks.

During training, visual stimuli were associated with their respective reinforcement and swapped pseudo-randomly between visits (see above). Experiments were balanced with respect to the association between a visual stimulus and a positive/aversive reinforcement. For each ant, the whole procedure (pre-training and training) lasted usually 2–3.5 h and took place always in the same day.

## Results

### Activity rhythms

We first monitored daily activity rhythms of *F. cunicularia* and *C. aethiops* in natural conditions. Weather temperature and illumination were recorded in parallel at both nest entrances. Figures 2A,B shows the mean activity rhythms (± S.E.) of *F. cunicularia* and *C. aethiops* (including the total number of ants and majors and minors, separately). While *F. cunicularia* started its activity early in the morning, between 05:00 and 06:00 h (Figure 2A), *C. aethiops* was already active at this time of the day (Figure 2B). *C. aethiops* majors had a relatively constant and low level activity throughout the day. In the case of *F. cunicularia* and *C. aethiops* minors, activity increased during the morning, but peaks of activity differed: while *F. cunicularia* exhibited maximal activity around 12:00 h, *C. aethiops* minors reached a maximum around 10:00 AM, after which activity dropped significantly. Thus, at the time when *F. cunicularia* was most active, which corresponded to a great increase in illumination levels (Figures 2C,D), *C. aethiops* minors were less active. Activity of *F. cunicularia* decreased progressively in the afternoon (from 15:00 to 20:00 h) while that of *C. aethiops* minors increased and extended beyond twilight into the night (from 20:00 to 01:00 h), during which higher numbers of ants were recorded (Figure 2B). At that time no *C. aethiops* majors were observed; yet the fact that some majors were found already at 05:00 h suggests that their activation occurred earlier, during the last hours of the night.

Thus *F. cunicularia* exhibited a diurnal activity rhythm that was significantly and positively correlated both with illumination (Figure 2C; Spearman rank correlation: ρ = 0.84; *p* < 0.0001) and temperature (Figure 2C; ρ = 0.38; *p* < 0.05). On the contrary, *C. aethiops* minors displayed a rhythm that was significantly and negatively correlated with illumination (Figure 2D; ρ = −0.59; *p* < 0.05) but not with temperature (Figure 2D; ρ = −0.21; NS). In the case of *C. aethiops* majors, the opposite trend was found: activity did not correlate with illumination (Figure 2D; ρ = 0.22; NS) but was inversely and significantly correlated with temperature (Figure 2D; ρ = −0.32; *p* < 0.05). Thus, whilst *C. aethiops* minors performed their activities during periods of lower illumination, majors were more active during periods of lower temperature. The fact that the activity of *C. aethiops* minors (and to a lesser extent of *C. aethiops* majors) occurred within both the light and dark portions of the day allows describing their rhythm as cathemeral (Tattersall, 1987). The segregated pattern of activity of both sympatric species may have evolved to avoid competition.

### Allometry and eye structure

We obtained morphometric measurements from both ant species. Measurements were taken separately for majors and minors of *C. aethiops* given their important allometric differences (Figure 1A). Such differences are absent in *F. cunicularia*. We used thorax length (Th_*l*_) as a measure of ant size and showed that the groups differed significantly [One-factor ANOVA: Th_*l*_: *F*_(2,27)_ = 93.77; *p* < 0.0001]. *F. cunicularia* ants had a significantly shorter thorax than both majors and minors of *C. aethiops* (Tukey test; *p* < 0.05 for both comparisons). *C. aethiops* minors, on the other hand, had a shorter thorax than *C. aethiops* majors (*p* < 0.001).

**Figure 3:**
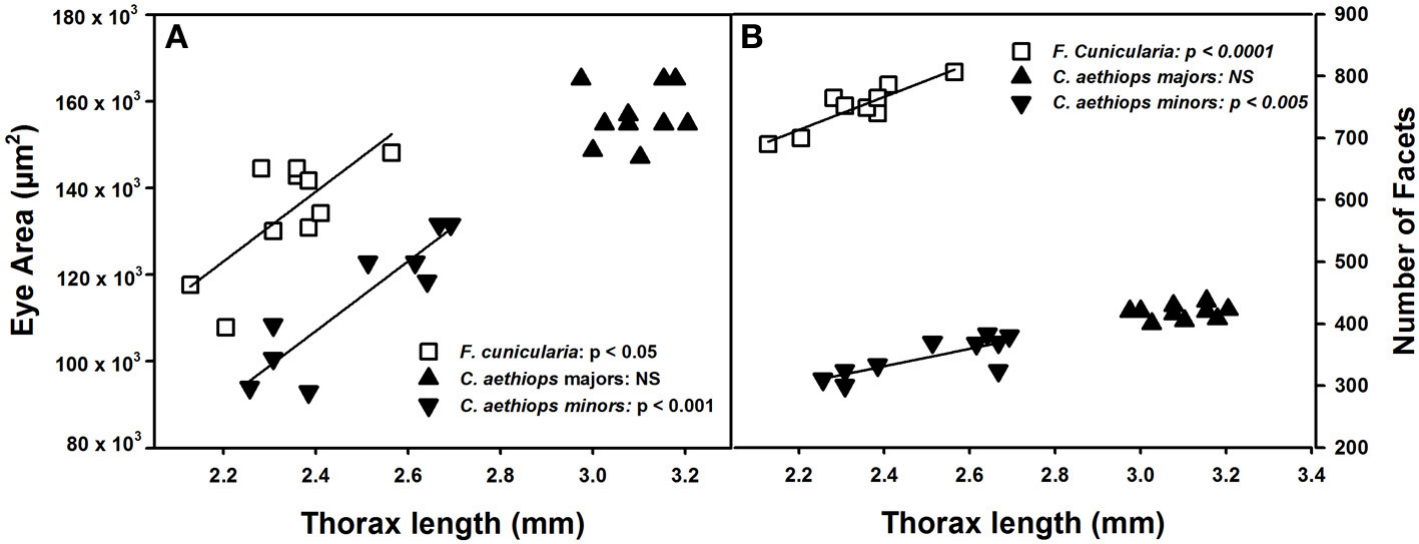
**Eye area (μm^2^) and number of facets as functions of thorax length (mm). (A)** Eye area increased significantly with thorax length in *F. cunicularia* and *C. aethiops* minors but not in *C. aethiops* majors. **(B)** The number of eye facets increased significantly thorax length in *F. cunicularia* and *C. aethiops* minors but not in *C. aethiops* majors. NS, not significant.

The surface area of compound eyes also varied significantly among the three ant groups [*F*_(2,27)_ = 28.39, *p* < 0.0001]. It was significantly larger in *C. aethiops* majors, smaller in minors, and of intermediate size in *F. cunicularia* ants (*p* < 0.05 for all comparisons). Figure 3A shows the relationship between eye area and thorax length, the parameter used to characterize ant size. Eye area increased with thorax length in *F. cunicularia* (*r* = 0.73, *p* < 0.05) and *C. aethiops* minors (*r* = 0.91, *p* < 0.001) but not for *C. aethiops* majors (*r* = 0.16, *p* = 0.66). Figure 3B shows the relationship between the number of eye facets and thorax length. The number of facets increased significantly with thorax length in *F. cunicularia* (*r* = 0.90, *p* < 0.0001) and *C. aethiops* minors (*r* = 0.79, *p* < 0.005) but not in *C. aethiops* majors (*r* = 0.20, *p* = 0.60).

**Figure 4:**
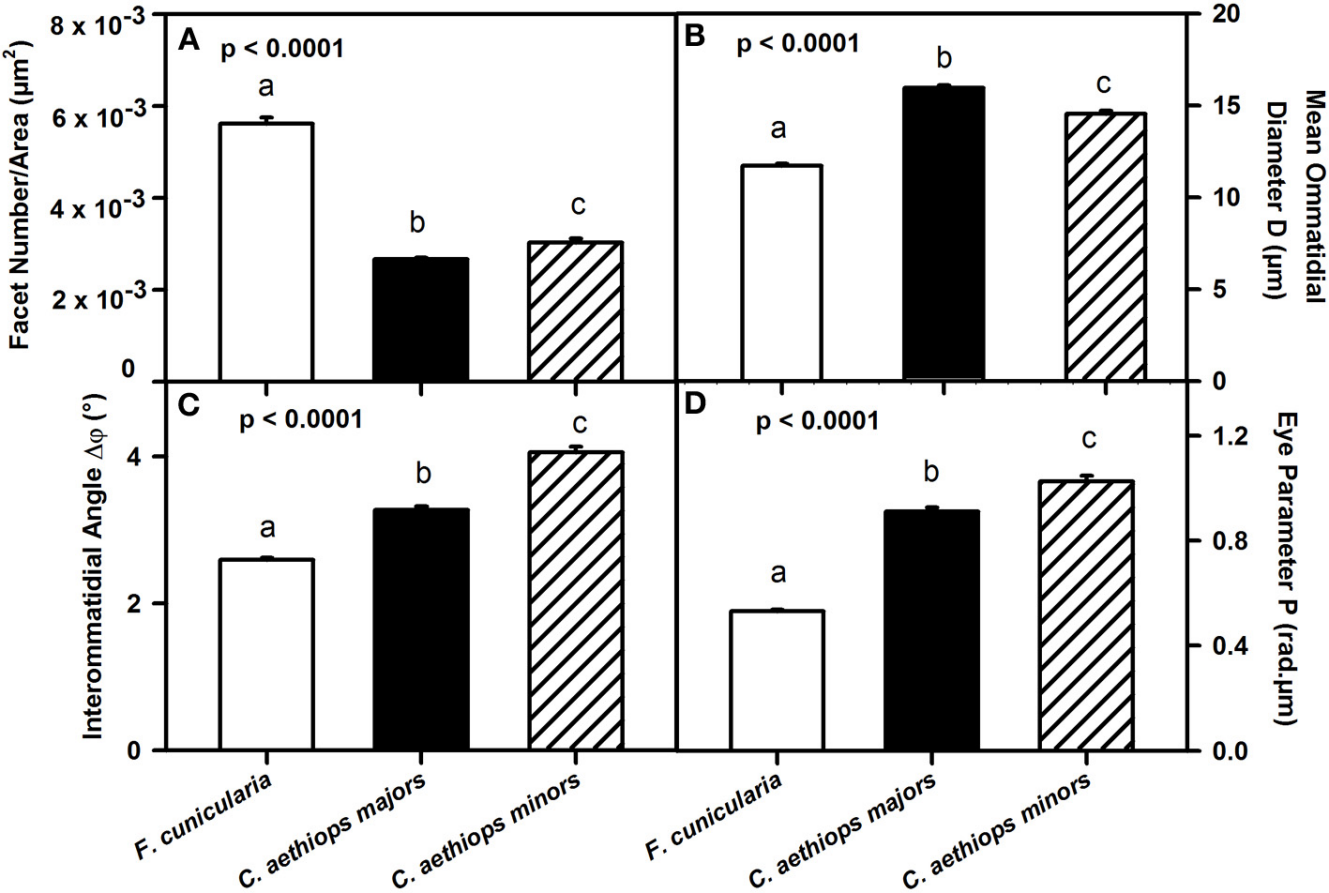
**Comparative analysis of eye parameters between *F. cunicularia* and *C. aethiops* majors and minors**. Different letters indicate significant differences. **(A)** Number of ommatidial facets per area (μm^2^) of compound eye, which provides an estimate of compound eye resolution. *F. cunicularia* exhibited significantly more facets per unit area of compound eye than both *C. aethiops* major and minors (*p* < 0.001 for both comparisons); *C. aethiops* minors had in turn a significantly higher number of facets per unit area than majors (*p* < 0.05). **(B)** Facet diameter **(D)** (μ m) differed significantly between the three groups of ants. It was smaller in *F. cunicularia*, intermediate in *C. aethiops* minors and larger in *C. aethiops* majors. All values differed significantly from each other (*p* < 0.001). **(C)** Interommatidial angle Δφ(°), which provides a measure of compound eye resolution and sampling density. *F. cunicularia* ants exhibit smaller interommatidial angles and thus highest resolution, while *C. aethiops* majors exhibit intermediate angles and thus intermediate resolution, and *C. aethiops* minors larger angles and thus lowest resolution. All values differed significantly from each other (*p* < 0.001). **(D)** Eye parameter P (rad.μm), which provides a measure of the sensitivity of insect eyes; *F. cunicularia* ants had smaller *P*-values, *C. aethiops* minors had larger *P*-values and *C. aethiops* majors had intermediate values. All three values differed from each other (*p* < 0.001).

We then referred facet number to the area of the compound eye (μm^2^) in order to obtain an estimate of the resolution of the compound eyes in the three ant groups (Figure 4A). Ants differed significantly in the number of facets per unit area (μm^2^) of compound eye [*F*_(2,27)_ = 299.66; *p* < 0.0001]. *F. cunicularia* had significantly more facets per unit area of compound eye than both *C. aethiops* major and minors (*p* < 0.001 for both comparisons); *C. aethiops* minors had a significantly higher number of facets per unit area than majors (*p* < 0.05).

To provide a finer analysis of the eye structure in the three groups of ants, we focused on the ommatidial diameter (D) and the interommatidial angle (Δφ) (see Figure 1B). The ommatidial diameter varied significantly between the three groups of ants [Figure 4B: *F*_(2,107)_ = 201.52, *p* < 0.0001]. Specifically, *F. cunicularia* ants had smaller ommatidial diameters (11.72 μm ± 0.10), *C. aethiops* majors, larger diameters (15.95 μm ± 0.13) and *C. aethiops* minors intermediate values (14.53 μm ± 0.19)(Tukey test; *p* < 0.001 for all three comparisons).

Interommatidial angles Δφ also provide a measure of compound eye resolution and sampling density (the smaller the interommatidial angle, the higher the potential resolution and sampling density of the compound eye). The three groups of ants differed significantly in Δφ [Figure 4C: *F*_(2,114)_ = 175.32, *p* < 0.0001], with *F. cunicularia* ants exhibiting smaller interommatidial angles (2.59° ± 0.03), *C. aethiops* majors intermediate angles (3.27° ± 0.05) and *C. aethiops* minors larger angles (4.05° ± 0.08). All values differed significantly from each other (*p* < 0.001 for all comparisons).

The eye parameter (P), which allows examination of the trade-off between sensitivity and resolution of a compound eye (Snyder, 1979), was obtained for all three groups of ants by multiplying the interommatidial angle value (Δφ) by the diameter of a single ommatidium (*D*). P varied significantly between ant groups [Figure 4D: *F*_(2,114)_ = 265.68; *p* < 0.0001]. *F. cunicularia* ants had smaller *P*-values (0.53 μm.rad ± 0.008), *C. aethiops* minors had larger *P*-values (1.03 μm.rad ± 0.02) and *C. aethiops* majors had intermediate values (0.91 μm.rad ± 0.02) but which were nevertheless close to those of *C. aethiops* minors. All values differed significantly from each other (*p* < 0.001 for all comparisons).

### Visual-discrimination performance

**Figure 5:**
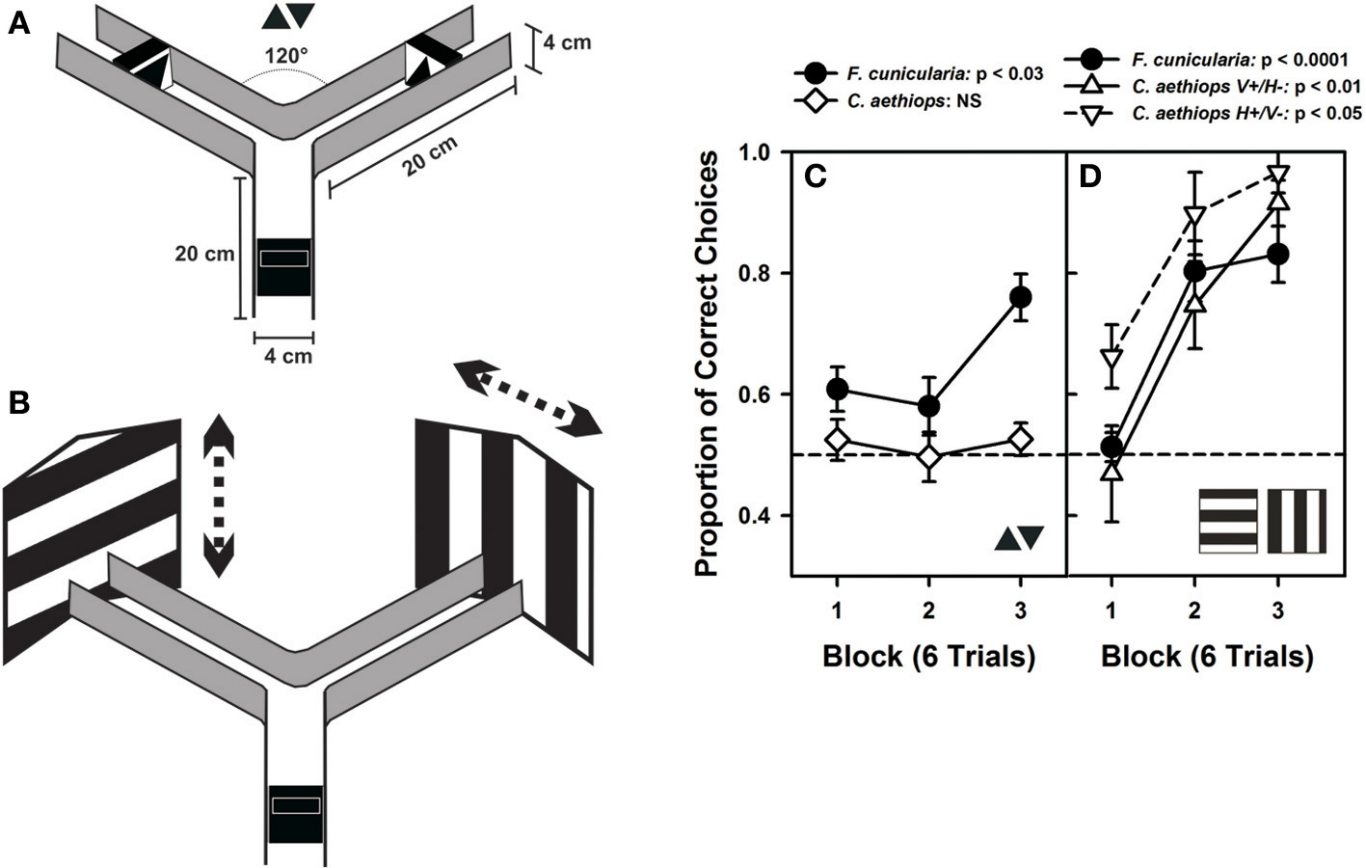
**Visual performances of *F. cunicularia* and *C. aethiops*. (A)** The Plexiglas Y-maze used for visual discrimination learning in ants. The maze presents the two small-field visual stimuli that ants had to learn to discriminate, a small black triangle pointing upward and another black triangle pointing downward, both presented on a white background. **(B)** Visual discrimination learning of two large-field visual stimuli in the form of two large black-and-white gratings, one horizontal and the other vertical, which exceeded the extent of the maze back walls. Gratings had stripes 8 cm in width and were randomly shifted (see dashed arrows) from visit to visit to prevent the exclusive learning of local cues. **(C)** Visual-discrimination learning of the two small-field visual stimuli. Proportion of correct choices along three consecutive blocks of six trials. *F. cunicularia* ants learned the visual discrimination while *C. aethiops* ants did not. **(D)** Visual discrimination learning of the two large-field visual stimuli. Proportion of correct choices along three consecutive blocks of six trials. Both ant species learned the visual discrimination. *C. aethiops* ants learned significantly better the discrimination in which the horizontal grating was rewarded and the vertical grating was non-rewarded than the reversed situation so that both experimental situations appear differentiated.

In a first visual-discrimination problem, ants were trained to discriminate two small-field black triangles, one pointing upward and the other pointing downward (Figure 5A). No difference in performance was found between the two groups of *F. cunicularia* ants conditioned to choose either the upright triangle or the inverted triangle as rewarded stimulus [*F*_(1,10)_ = 1.82; *p* = 0.21] so that results were pooled and treated as a single group (Figure 5C). *F. cunicularia* ants learned to discriminate the two triangles during the three blocks of training [*F*_(2,22)_ = 4.52; *p* < 0.03], in particular from the second to the third block of trials (Tukey test: *p* < 0.03). On the contrary, *C. aethiops* trained with the same discrimination problem, and whose results could also be pooled [*F*_(1,10)_ = 0.00; *p* = 0.99], were incapable of learning this discrimination under the same experimental conditions [Figure 5C: *F*_(2,22)_ = 0.21; *p* = 0.82]. As a consequence, the performance of both species differed significantly in this discrimination task [*F*_(1,22)_ = 29.01; *p* < 0.0001]. Increasing the number of conditioning trials in the case of *C. aethiops* did not yield any improvement in discrimination (not shown).

In a second discrimination problem, ants were trained to discriminate two large-field patterns whose extent surpassed largely the limits of the maze (Figure 5B); the patterns consisted of two large black and white gratings, one vertical, and the other horizontal. Gratings were shifted randomly from visit to visit to force ants to focus on global and not local cues. Vertical gratings were shifted either left- or rightward while horizontal gratings were shifted up- or downward. *F. cunicularia* ants behaved similarly irrespective of whether the rewarded grating was horizontal or vertical [*F*_(1,10)_ = 1.34; *p* = 0.27], so that results of both subgroups were pooled and treated as a single group. *F. cunicularia* ants learned to discriminate the two large stimuli during the three blocks of training [Figure 5C: *F*_(2,22)_ = 21.50; *p* < 0.0001]. *C. aethiops* ants showed differences depending on whether the vertical or the horizontal grating was rewarded [*F*_(1,9)_ = 18.09; *p* < 0.03], so that their results were not pooled. In particular, discrimination learning was better when the rewarded pattern was the horizontal grating (Figure 5C). Despite this difference, both subgroups of *C. aethiops* learned the discrimination between the two large-field stimuli during the three blocks of training [Figure 5C: vertical rewarded/horizontal non-rewarded: *F*_(2,10)_ = 8.46; *p* < 0.01; horizontal rewarded/vertical non-rewarded: *F*_(2,8)_ = 8.14; *p* < 0.05]. Thus, all three groups (*F. cunicularia* and the two subgroups of *C. aethiops*) learned efficiently to discriminate the vertical from the horizontal edge patterns; discrimination was better in *C. aethiops* trained with the horizontal grating rewarded [*F*_(2,20)_ = 5.09; *p* < 0.05].

## Discussion

Our findings highlight the adaptive interplay between the optic properties of the compound eye, the visual environments, and circadian activity, and the visual-discrimination capabilities of ants. Diurnal *F. cunicularia* ants exhibit high optic resolution based on more ommatidia, smaller facet diameter, smaller interommatidial angles, and a small P parameter value. These features enabled them to learn both small-field and large-field pattern discriminations. By contrast, *C. aethiops* minor ants, which are active during twilight and night and thus exhibit an inverse correlation between their activity and illumination levels (Figure 2), favor light capture and exhibit larger interommatidial angles and a higher P parameter value, consistently with optic strategies of other nocturnal insects (McIntyre and Caveney, 1998; Warrant et al., 2004; Greiner, 2006; Somanathan et al., 2009). As a consequence, these ants only learned large-field but not small-field pattern discriminations. In *C. aethiops* majors, we found higher facet diameters and intermediate interommatidial angles and *P*-value (Figure 4). These intermediate values may result from the compromise between a larger body size and a low level of activity outside the nest, which determined a lack of correlation between activity and illumination (Figure 2). Due to this low activity, and the impossibility of ensuring a high appetitive motivation, the visual discrimination performances of *C. aethiops* majors were not quantified in this work.

The optical differences found between *F. cunicularia* and minors of *C. aethiops*, which reflect their differences in activity and visual niche, are consistent with results on optical specializations of compound eyes in diurnal and nocturnal insects. For instance, in a study on 15 different Apoidea species, nocturnal foraging bees had larger compound eyes and facets than diurnal ones (Jander and Jander, 2002). Similar results were found in ants: in a study on the relationship between the timing of nuptial flights of 10 species of leaf-cutter ants *Atta* sp. and the morphometry of their compound eyes, species flying during the night had significantly larger facets than species flying during the day (Moser et al., 2004). Also, in four congeneric sympatric species of *Myrmecia* ants, eye area, facet size and facet numbers increased from day- to night active species (Narendra et al., 2011).

Although larger insects usually benefit from a high spatial resolution acquired through a higher number of ommatidia and smaller interommatidial angles (Spaethe and Chittka, 2003; Kelber et al., 2006; Somanathan et al., 2009), the specializations imposed by diurnal/nocturnal life styles may modify this trend. For instance, the smaller and diurnal *F. cunicularia* ants had the highest spatial resolution (smaller interommatidial angles; see Figure 4C) consistently with their finer pattern-discrimination capabilities (Figure 5C). On the contrary, cathemeral *C. aethiops* sacrificed resolution (larger interommatidial angles; see Figure 4C) in favor of light capture (see Figure 4B for ommatidial diameter). Similar trends were found in sympatric species of *Myrmecia*, where the purely day active *M. croslandi* had the smallest facet lens, followed by the diurnal/crepuscular *M. tarsata*, while lens diameter was largest in the crepuscular/nocturnal species *M. nigriceps* and *M. pyriformis* (Greiner et al., 2007).

The trade-off between sensitivity and resolution of a compound eye is usually evaluated by means of the eye parameter P, which relates the mean interommatidial angle (Δφ) and the mean ommatidial diameter (D) (Snyder, 1979). Diurnal insects active in bright light usually exhibit smaller *P*-values reflecting an optimization of visual resolution. The fact that *F. cunicularia* ants, active at highest light intensities, presented the lowest *P*-values (0.53 μm.rad) is consistent with this trend. *P*-values predicted for nocturnal insects are higher (greater than 2 μm.rad; see Snyder, 1979). In our case, *C. aethiops* ants exhibited lower *P*-values of 1.03 and 0.91 μm.rad for minors and majors, respectively, which may be related to the fact that these insects were not purely nocturnal but distributed their activities during night and day.

As mentioned above, optic differences have been reported for other sympatric insect species that are phylogenetically related and that have adopted different lifestyles (Greiner et al., 2007; Gonzalez-Bellido et al., 2011; Narendra et al., 2011). Yet, here we show for the first time that these differences translate into distinct visual discrimination performances when insects are tested in a common experimental design in controlled laboratory conditions (Figure 5). Small-field visual stimuli, for which shape discrimination and thus local sampling of pattern details are necessary, was achieved by the diurnal *F. cunicularia* owing to its better optic resolution, and not by the cathemeral *C. aethiops* minors. However, when visual discrimination involved large-field visual stimuli (the large contrasting gratings employed in our experiments), both species were capable of mastering the discrimination. Despite a lower optic resolution, *C. aethiops* minors can use their increased light sensitivity to distinguish between differently oriented large-extent black and white areas and thus achieve the discrimination task. Differences in illumination, which are normally experienced by these species owing to their different activity rhythms, were ruled out in these experiments as insects were all studied under the same intermediate light conditions. Thus, the differences in discrimination power exhibited by *F. cunicularia* and *C. aethiops* were intrinsically determined by the properties of their respective visual systems. These differences did neither result from environmental factors nor from differences in motivation/circadian activity, as experiments were performed at times of the day in which levels of activity were comparable for both species.

Our work highlights the complex interactions that exist between visual niche exploitation, circadian activity, compound-eye structure, and visual-discrimination capabilities. Additional factors also might be similarly adapted to visual niche and light conditions; for instance, screening-pigment physiology within ommatidia, as well as phototransduction speed or crosstalk between photoreceptors within an ommatidium, could also evolve to optimize information processing and adaptive responses to ecological demands (Gonzalez-Bellido et al., 2011). In day-active ants, radial migration of retinula cell screening pigments has been reported as a light adaptation mechanism in which the pigments surround the rhabdom in the light-adapted state and move away from the rhabdom in the dark-adapted state (Brunnert and Wehner, 1973). Such a mechanism could be available in *F. cunicularia*. The case of *C. aethiops* could be more similar to that of the Australian intertidal ant *Polyrhachis sokolova*, which is active during low tides at both day and night and thus experiences a wide range of light intensities. A recent study on this species has shown that it has developed an extreme pupillary mechanism during which the primary pigment cells constrict the crystalline cone to form a narrow tract of 0.5 μm wide and 16 μm long. This pupillary mechanism protects the photoreceptors from bright light, making the eyes less sensitive during the day (Narendra et al., 2013; see also Menzi, 1987). A similar mechanism may be available in *C. aethiops* ants, which also face a wide range of light intensities due to their cathemeral activity rhythm. Furthermore, nocturnal ants have typically large rhabdoms, which increase the amount of photons that can be captured, thus increasing visual sensitivity under low illumination (Greiner et al., 2007); it is, therefore, possible that *C. aethiops* ants possess rhabdoms that are larger than those of *F. cunicularia* to cope for the dim light conditions under which the former, but not the latter, are active. Moreover, visual processing at the central level (i.e., temporal and spatial integration) also could differ between sympatric species in order to cope efficiently with the visual environment.

All in all, our results reveal the sophisticated interplay between compound-eye optics, visual niche, activity rhythms, and visual discrimination capabilities in ants. They reaffirm that behavioral and morphological traits respond to ecological pressures and lifestyle and underline the relevance of an ecological/evolutionary framework for analyses in behavioral neurosciences.

### Conflict of interest statement

The authors declare that the research was conducted in the absence of any commercial or financial relationships that could be construed as a potential conflict of interest.
